# Quality Analysis of YouTube Videos Presenting Pelvic Floor Exercises after Prostatectomy Surgery

**DOI:** 10.3390/jpm11090920

**Published:** 2021-09-15

**Authors:** Alvaro Manuel Rodriguez-Rodriguez, Maria Blanco-Diaz, Pedro Lopez-Diaz, Marta de la Fuente-Costa, Maria Cruz Sousa-Fraguas, Isabel Escobio-Prieto, Jose Casaña

**Affiliations:** 1 Exercise Intervention for Health Research Group (EXINH-RG), Department of Physiotherapy, University of Valencia, 46010 Valencia, Spain; alvaro.manuel.rodriguez@gmail.com (A.M.R.-R.); jose.casana@uv.es (J.C.); 2 Physiotherapy and Translational Research Group (FINTRA-RG), Institute of Health Research of the Principality of Asturias (ISPA), Faculty of Medicine and Health Sciences, University of Oviedo, 33006 Oviedo, Spain; lopezdpedro@uniovi.es (P.L.-D.); fuentemarta@uniovi.es (M.d.l.F.-C.); sousamaria@uniovi.es (M.C.S.-F.); 3 Departamento de Fisioterapia, Facultad de Enfermería, Fisioterapia y Podología, Universidad de Sevilla, 41009 Sevilla, Spain; iescobio@us.es

**Keywords:** YouTube, prostate cancer, healthy behavior, exercises, pelvic floor, urinary incontinence

## Abstract

Background: Prostate cancer (PC) is a major cause of disease and mortality among men. Surgical treatment involving the removal of the prostate may result in temporary or permanent erectile dysfunction (ED) and urinary incontinence (UI), with considerable impact on quality of life. Pelvic floor muscle training (PFMT) is one of the recommended techniques for the prevention, treatment, and rehabilitation of postoperative complications. The aim of this observational study was to assess the quality of YouTube videos—accessible to any patient—related to exercises after prostatectomy surgery. Methods: A systematic search was performed on YouTube on 24 September 2020. One hundred and fifty videos were selected and analyzed. Two statistical analyses were conducted based on machine-learning techniques, and videos were classified as ‘Relevant’ or ‘Non-Relevant’ using Principal Component Analysis (PCA) models. Two reviewers conducted independent analyses. Inter-observer agreement and individual correlations of video data were evaluated with the Intraclass Correlation Coefficient (ICC). Information quality, reliability, and accuracy were measured using the DISCERN Scale and Global Quality Score (GQS), while video popularity was evaluated using the Video Power Index (VPI). Results: DISCERN scored a mean of 3.35 and GQS scored 3.38. Average number of views was 124,354, mean duration was 14:42 min, mean days online was 1777, mean view ratio was 138.30, mean Likes was 1082, mean Dislikes was 68.58, and mean VPI was 92.28. Conclusions: The quality of the videos available on YouTube regarding the recommended pelvic floor exercises in PC surgery, according to the scores obtained, is High. Educational and health institutions, health professionals, government health authorities, and policy makers need to be involved in the proper development of policies to improve the information available on the web in order to have a positive impact on the healthy behavior of the population.

## 1. Introduction

According to Globocan 2020, prostate cancer is the fourth most common cancer overall, with an incidence of 1,414,259 in men in 2020, exhibiting higher incidence in developed countries.

Prostate cancer is a major cause of disease and mortality among men, with 375,304 men dying of it each year [1,2].

Radical prostatectomy (RP) is a common curative treatment to prevent metastasis. Although mortality after RP is low (5-year survival: 95%), morbidity is high [3]. Surgical treatment involving the removal of the prostate may result in temporary or permanent erectile dysfunction (ED) and urinary incontinence (UI), with considerable impact on quality of life (QoL) [4,5,6,7]. While the postoperative incontinence rate is 1% in patients undergoing prostatectomy for benign reasons, a rate between 2% and 66% has been reported after RP [7]. Depending on how continence is defined, almost 80% of men experience incontinence after RP [3]. ED affects 26% to 100% of patients after RP, and the main cause is known to be injury to the neurovascular bundles [5].

Treatment of incontinence involves noninvasive behavioral therapeutic methods consisting of diet modification, bladder training, pelvic floor muscle exercises (PFME), biofeedback, and functional electrical stimulation [4]. Pelvic floor exercises have been used to improve urinary continence following RP, with good results [5,8]. Urinary continence can be achieved through contraction training of the pelvic floor muscles [6]. Pelvic floor muscle training (PFMT) is one of the recommended techniques for the prevention, treatment, and rehabilitation of RP-related complications [5]. It can improve UI and ED after prostatectomy [6]. The aim of PFME, first defined by Arnold Kegel in 1948 as a behavioral therapeutic method for treating incontinence, is to enhance muscle volume and contraction strength in case of increased intra-abdominal pressure [4]. Many studies of PFMT have been conducted in post-prostatectomy patients, delivered both before and after surgery. An increase in speed of recovery was found in more active rehabilitation intervention targeting physiological PFM function of fast- and slow-twitch muscle fibers [9]. However, a major barrier to the success of any training program is adherence, and PFMT is no different. Many patients search the Internet for medical information, but they lack the tools to evaluate the advice provided [10]. As of 3 March 2020, an estimated 58.7% of the global population, representing 4,574,150,134 users, had access to the Internet [11]. YouTube is one of the most popular Internet sites and it also operates as a primary Internet platform for consumer-targeted health information. It is the largest video archive website in the world and attracts 95% of Internet users, with 30 million active users every day [12]. It has 5 billion visits per day and 1 billion hours are watched daily [13]. YouTube and other online video-sharing sites have also become important channels for science popularization and communication [14].

Advances in eHealth technology have cultivated transactional opportunities for patients to access, share, and monitor health information [15]. Digital behavior change interventions use digital health technologies for behavior modification for the maintenance and improvement of health [16], and patients and health professionals tend to search the Internet for information about many health-related topics. In fact, 81% of all Internet users go online and search for information related to health [13]. Generally, patients search for detailed information about recommended exercises. Therefore, the authorship, quality, and validity of the information contained in videos must be considered [12], as some of them present commercial content that may affect the attitude and decision-making of consumers [17].

Video-sharing websites must be understood as social media sites where there is no editorial selection or quality assessment. Likewise, potentially harmful and inaccurate information about science and biomedical topics can be disseminated. The kind of content stored on YouTube and the quality of this information is unclear [18], but a platform such as YouTube has the potential to be an important resource for sharing and disseminating health-related information [19].

One question that has been highlighted by biomedical institutions is whether YouTube provides users and (potential) patients with accurate and helpful information or might the videos possibly be harmful and misleading. Healthcare professionals and organizations should be encouraged to provide more beneficial material and animated videos to people looking for comprehensive, reliable information on the Internet [20]. Healthcare providers and government agencies have expressed their concern about the veracity and quality of the information available on this platform as the health-content videos uploaded by various sources can be misleading and present inaccurate information to patients [20].

## 2. Materials and Methods

### 2.1. Search Strategy

On 24 January 2021, a search was conducted on http://www.youtube.com (assessed on 24 January 2021), using the following search terms: ‘Prostate cancer–exercises–pelvic floor’.

**Figure 1:**
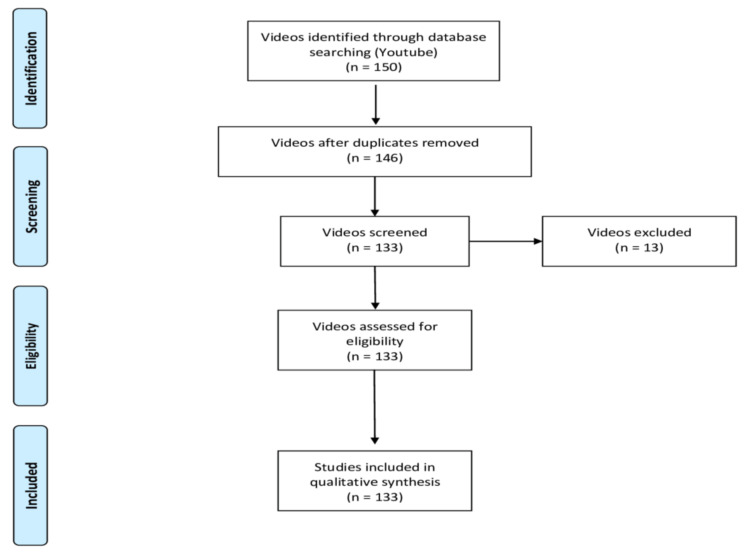
Video flow diagram [21].

The first 150 videos available to viewers were selected. The aim was to replicate a simple search strategy that could be conducted by anyone, so the search was not restricted using filters. Hence, YouTube sorted video results by their relevance according to the patented ranking algorithm active on that specific day. All the videos were added to a spreadsheet and then submitted for screening for duplicates, as well as in order to apply the research team’s inclusion and exclusion criteria. Exclusion criteria were non-English language, duplicated videos, pelvic floor exercises for women, and/or related to advertisements. Finally, 133 videos were assigned to 2 different examiners who viewed, analyzed, and evaluated them independently over a period of 5 weeks (Figure 1).

### 2.2. Outcome Measures

Based on their production source, the videos were categorized into six groups: Health Organization (clinic/hospital), Healthcare Professional, Non-Healthcare Professional, Academic Institution, Media (TV, newspaper, etc.), or NGOs. Videos were also coded according to their continent of origin (America, Europe, Asia, Africa, Australia).

Information on when the exercises were recommended (preoperative, postoperative, or both) and the name of the exercises themselves (pelvic floor exercises, Kegel exercises, breathing exercises, yoga exercises, general exercises, or various) was also collected.

The objective of the videos was also identified, based on their indication with regard to UI, fecal incontinence (FI), sexual disfunction (SeD), pelvic pain (PP), and/or incontinence + sexual disfunction (UI + SeD). The quality of the information provided was completed with other collected variables, such as patient position for performing the exercises, number of daily and weekly repetitions, fiber type (phasic or tonic), breathing type, warning about the inappropriate use of accessory muscles, and whether or not it was necessary to use some kind of material to carry out the exercises.

Similarly, descriptive characteristics of each video (View counts, Likes, Dislikes, Origin, Days online, Author, and Duration) were collected. Video popularity was assessed using the Video Power Index (VPI) ((like count/(dislike count + like count)) × 100)) [12,22,23] and View Ratio (Views count/Days online) [24]. The educational quality of the 133 selected videos was determined using the DISCERN tool (Quality Criteria for Consumer Health Information) [25] and the Global Quality Scale (GQS) [26].

A modified 5-point DISCERN tool [27], adapted from the original DISCERN tool for assessment of written health information by Charnock et al. [25], was used for this study. It was created by the Division of Public Health and Primary Care at Oxford University, London, to gauge the quality of information regarding treatment choices for health problems, and was first published in 1999 [25]. The questionnaire consists of a total of 5 questions in addition to an overall quality rating. Each question represents a different quality criterion, rated from 1 to 5 points (1: Very Poor, 2: Poor, 3: Average, 4: High, and 5: Very High quality).

GQS assesses the content quality of online resources. One point is assigned for each of the five identifiable criteria present in a video, with five being the highest educational quality [26]. This scale covers the accessibility and quality of the information, the overall flow of information, and how useful it would be for a user [24].

### 2.3. Statistical Analysis

Two different analyses were performed on the dataset: The first analysis was based on machine learning techniques, considering a binary classification problem, and thereby dividing the sample into ‘Relevant Videos’ (C1) or ‘Non-Relevant Videos’ (C2). These two classes were defined in two cases, using the means of the DISCERN and Principal Component Analysis (PCA) [28]. PCA visualizes the information in a dataset described by multiple interrelated variables. The information in a given dataset corresponds to the total variation. PCA identifies directions (or principal components) along which there is a maximum variance in the data. It is used to extract the important information from a multivariate dataset and express this information as a set of a few new variables, i.e., the principal components. Thus, PCA reduces the dimensionality of multivariate data to two or three principal components (in this case, two principal components were used to group data) that can be visualized graphically, with minimal loss of information.

The objective of the first analysis was to determine the variables that best distinguish these two video classes. The classification problem was studied in two cases, when both classes were defined by the following variables:

1. DISCERN: In this case, samples in the first class, C1 (Relevant Videos), were considered to be the videos whose DISCERN variable value was greater than the mean for this variable, and the rest of the videos were defined as C2.
2. PCA: This was a binary variable that already contained the class of each sample after performing a PCA and a k-means algorithm. In this case, the sample grouping into C1 and C2 is given by the variability in data and there is no ‘a priori’ information as to the relevance of the videos in each class. Consequently, reducing the number of variables to two (as per PCA) that group the most important information of all the initial variables means that no information is lost.

For each of the two cases, a classification algorithm was applied to attain a minimum list of variables that best assigned the samples into the two groups. Accuracies were determined by Leave-One-Out Cross Validation (LOOCV) using a nearest-neighbor classifier. The discriminatory power of the variables was established according to their Fisher’s Ratio (FR). Variables with an elevated FR are highly discriminatory, since they have low intraclass dispersion and high interclass distance. In a binary classification, the FR of the variable *j* is given by:

$$\frac{{\left(\mu_{j1}-\mu_{j2}\right)}^{2}}{\sigma_{j1}{}^{2}+\sigma_{j2}{}^{2}}$$

where $\mu_{ji}$ is a measure of the center of mass of the probability distribution of the variable *j* in class *i* (*i* = 1, 2), and $\sigma_{ji}$ is a measure of its dispersion within this class. Variables with a high FR are highly discriminatory, since they have low intraclass dispersion and high interclass distance.

In addition to FR, Pearson, Kendal, and Spearman Correlation Factors of the variables with the defined classes were computed. These factors also reveal the relevance (discriminatory power) of the variables for the classification criteria.

The second analysis was a statistical one using a t-test and Wilcoxon test, illustrating how significant the differences between both classes are from the perspective of each variable. The statistical analysis also shows the relevance of each variable regarding the grouping of the samples into C1 and C2, in each of the two cases. The *t*-test determines whether the means of the variables in both datasets are significantly different from each other. The null hypothesis (H0) assumes that there is no difference between groups. When the statistical analysis yields 1, it means that there is enough evidence to reject this hypothesis, and if it results in 0, the null hypothesis must be accepted. The accepted value level of significance or error probability is alpha < 0.05. Consequently, the Wilcoxon test essentially calculates the difference between each set of pairs and analyzes these differences, whereas H0 refers to the equality of the population medians of two groups of samples (C1 and C2).

## 3. Results

### 3.1. Statistical Analysis

Descriptive statistics were obtained from each video, calculating their mean and establishing their minimum and maximum values along with their standard deviation (SD). The same values were obtained for the quality scales.

An ICC analysis was conducted to gauge inter-examiner concordance, with 95% confidence intervals (CI) based on mean rating (k = 2), consistency, two-way random model, and Pearson’s Correlation method. Level of significance was set at *p* < 0.05. Inter-rater agreement scored 0.9505 for this study.

### 3.2. Video Characteristics

**Figure 2:**
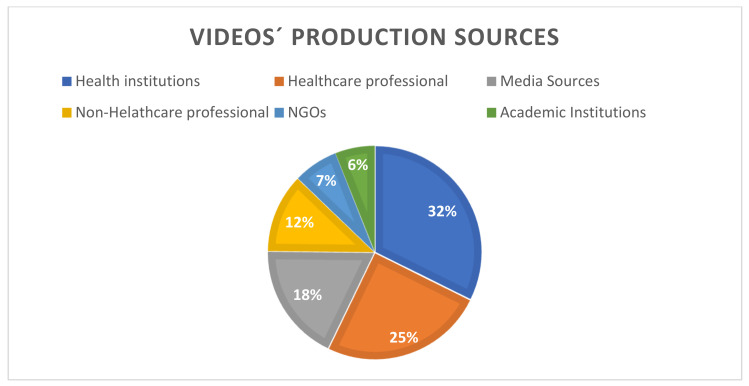
Video production sources.

Most of the videos (32.3%) were produced by Health Institutions, followed by Healthcare Professional (24.8%), Media sources (18.0%), Non-Healthcare Professional (12.0%), NGOs (6.8%), and Academic Institutions (6.0%) (Figure 2).

The origin of the videos was America in 65.4% of cases, Australia (28.6%), Europe (3.8%), Asia (0.8%), and Africa (0.8%).

The mean number of views was 124,354 (SD = 472,419), mean video duration was 14.42 min (SD = 19.34), mean number of days online was 1777 (SD = 1180), mean view ratio was 138.30 (SD = 788.31), mean Likes count was 1082 (SD = 4883), mean Dislikes count was 68.58 (SD = 265.45), and mean VPI was 92.28 (SD = 8.89). DISCERN and GQS scores of both independent observers were averaged to calculate the mean scores of each video. Considering average results, DISCERN scored a mean of 3.35 (SD = 1.25), while GQS scored a mean of 3.38 (SD = 1.02). Table 1 shows the basic descriptive statistics data and DISCERN and GQS results of the videos included.

According to the mean DISCERN scores of both observers, the quality of the videos was found to be Very Poor in 5.3% of the cases, Poor in 24.1%, Average in 21.1%, High in 27.8%, and Very High in 21.8%. However, referring to the mean GQS, the quality of the videos was found to be Very Poor in 3.8% of the cases, Poor in 17.3%, Average in 27.1%, High in 40.6%, and Very High in 11.3%.

As for the relationship between the production source and educational quality of the videos, according to DISCERN, the videos made by NGOs attained higher scores (4.33), followed by Health Institutions (3.88) and Academic Institutions (3.88), Healthcare Professional (3.45), Media (2.79), and finally those created by a Non-Healthcare Professional (1.75). Likewise, the GQS (the other variable that alludes to educational quality) ranked the origins in the same order: NGO’s and Academic Institutions also attained higher scores (4.00), followed by Health Institutions (3.77), Healthcare Professional (3.45), Media (2.96), and, finally, Non-Healthcare Professional (2.19). These data and the views ratio can be seen in Table 2.

The same table shows that higher scores on the DISCERN scale were assigned to videos from Australia (3.55), followed by Europe (3.40), America (3.33), Asia (3.00), and Africa (0.00). Meanwhile, the GQS scale assigned almost the same scores in videos made in Australia (3.55), followed by Europe (3.40), America (3.36), Asia (3.00), and, finally, Africa (1.00).

VPI mean scores for each of the five categories in which the DISCERN and GQS variables classified the videos were also analyzed. Videos with a Very Poor DISCERN score had a VPI mean of 89.20, videos classified as Poor had a VPI of 92.75, Average videos had 92.11, High videos had 89.13, and videos classified as Very High for DISCERN had 94.83. Likewise, videos with Very Poor GQS score had a VPI mean of 89.88, videos classified as Poor had a VPI of 90.94, Average videos had 93.31, High videos had 90.30, and videos classified as Very High for GQS had 96.41.

Regarding the objective of the videos, 39.85% of them did not specify their objective, 26.32% aimed to treat UI + SeD, 23.31% UI, 5.26% SeD, 4.51% PP, and 0.75% FI.

The terms used by the authors to denominate the exercises in the videos were Pelvic Floor Exercises (PFE) in 42.11% of cases, followed by Kegel Exercises (25.56%), Undefined (17.29%), and General Exercises (15.04%).

To carry out the exercises, most authors did not indicate the need for specific complimentary materials (65.4%), 24.1% suggested some type of domestic material, and only 10.5% of videos indicated the use of specific material, such as balls, special benches, or gym equipment.

Regarding the period of time over which the authors recommended doing the exercises, most (48.1%) did not specify it, followed by 30.1% who recommend the exercises for the postoperative period, 18.0% for the pre- and post-operative period, and, finally, 3.8%, for the preoperative one.

Of the videos, 78.9% did not specify the frequency of performing the exercises, while 16.6% suggested a frequency of three times a day, 3.8% indicated once a day, and 0.8% recommended two times a day.

The position of the patient during the performance of the exercises was not specified in 72.9% of the videos, in 15% various positions were indicated, while only 6.0% of videos recommended doing them sitting down, supine in 5.3%, and standing up in 0.8% of cases. Regarding breathing during exercises, 91.7% of videos did not mention it, 5.3% referred to breathing but did not indicate how to do it, while 3.0% explained that it was to be done during exhalation. Finally, 94% of videos did not indicate anything about the use of accessory muscles, whereas 6% of them recommended not using them. Regarding the effort on different types of muscle fibers, 21.8% of videos recommended the work on the tonic fiber and 9.77% the phasic fiber. The rest of the videos made no recommendation concerning this topic.

## 4. Discussion

This study was undertaken to better comprehend the nature of evidence independently accessed by patients on a massive online, media-sharing platform. The increasing use of new technologies and vast amount of content available have turned the Internet into an important source of health information, for healthcare professionals (who use new technologies more frequently to communicate with patients and achieve the necessary adherence and interaction in health processes) as well as patients (who use it as a source of information). However, this entails some risk, in as much as the information available on the web is not subject to any kind of inspection. Consequently, this can lead to erroneous and even harmful messages being conveyed to users and/or viewers. In the search process to improve health processes, rigorous education and dissemination are necessary, preferably carried out by health professionals. In the case of the videos analyzed, most (32.3%) were produced by Health Institutions, which can be considered as a guarantee, due to the fact that they are in the second place of the quality scales.

Very few videos were produced by NGOs (6.8%), even though the quality of their productions obtained the highest scores, which is in agreement with Masefield et al., who observed that NGO data constitute a vast and valuable source of information for health policy makers and public health systems research [29]. Videos whose source of production is Non-Health Professionals are those that obtained the worst scores on these scales.

Unfortunately, the percentage of videos made by Academic Institutions was the lowest (6.0%), a result that is in line with other studies that claim that Health Institutions are underrepresented in the publication of videos of medical information [22]. Taking into account the fact that the quality of the videos that they produce is one of the highest, they should consider academic projects to increase their presence and flood the Internet with high-quality information. As in similar studies, it was difficult in many cases to find out who had developed the video content, as well as for viewers to assess the reputation of the sources [30].

The average length of the videos in our study was 14.42 min, which is higher than previous studies that reported an average duration of 6.17–10.35 min [22,31]. No statistically significant relationship was found between video length and their quality or popularity, contrary to the study by Akif Aydin that found that the videos with the longest duration and the highest VPI appeared to be associated with higher quality scores [23].

ICC is a widely used test–retest, intra-rater, and inter-rater reliability index. Based on the 95% confident interval of the ICC estimation [32], the inter-reviewer agreement for this study was 0.9505, pointing toward Excellent concordance between both examiners, according to the ICC intervals shown in Table 3.

PC incidence rates are highly variable worldwide. Research has shown that African American men have the highest incidence of prostate cancer worldwide [33], while Chu et al. [34] reported that incidence rates of prostate cancer were as much as 40 times higher among African American men than those in Africa. In 2018, the highest mortality rates were recorded in Central America (10.7 per 100,000 people), followed by Australia and New Zealand (10.2), and Western Europe (10.1) [33]. As found in this study, most videos were produced in America (65.4%), where, in 2021, the estimated number of new cases was 248,530 [2], followed by those produced in Australia (28.6%), which points towards countries with higher incidence and mortality as those that produce more videos about PC. The overall means of DISCERN and GQS indicate that the YouTube contents analyzed have medium–high reliability and quality. They also show higher scores for videos produced in Australia, Europe, and America.

A statistically significant relationship between DISCERN and GQS scales was also detected (Pearson Coefficient = 0.9023; *p* < 0.001). Analyzing the scores obtained to appraise the educational quality of videos with both scales, it should be noted that most videos were of High quality (27.8% and 40.6% of cases, for DISCERN and GQS scales, respectively), Very High in 21.8% (DISCERN) and 11.3% (GQS), Average quality was found in 21.1% (DISCERN) and 27.1% (GQS), Poor quality in 24.1% (DISCERN) and 17.3% (GQS), and Very Poor educational quality in 5.3% (DISCERN) and 3.8% (GQS) of cases. This contrasts with previous studies that identified a worrying amount of poor-quality and erroneous information in YouTube videos related to health [17,23,24]. The results shown in this study indicate, as other authors have already mentioned, that YouTube is a solid and useful source of information [35], and different ways are needed to ensure that users access the most appropriate ones.

Since there is no mark to identify high-quality videos, it is recommended for their easy identification that patients attend to the source of production. Since Academic Institutions, NGOs, and/or Health Institutions show higher figures in quality scales, these should be the reference. It would also be advisable to discard those videos that contain commercials and could present any conflict of interest.

**Figure 3:**
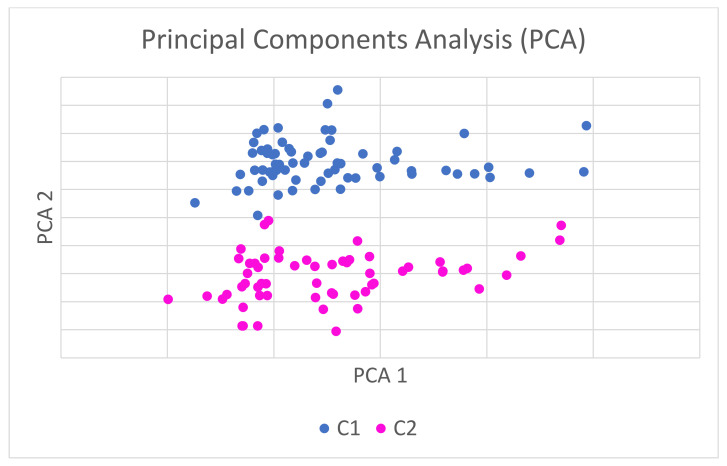
Extraction method: Principal Axis Factorization. Rotation method: Varimax Rotation with Kaiser Normalization.

From the perspective of the PCA, if the coordinates of each video are graphically represented in a coordinate system in which the axes refer to the principal components, the videos are naturally grouped into two groups of points, without any manual intervention or possible bias. These two groups, which can be seen in Figure 3, collect the videos with higher ratings in DISCERN and GQS in one group (C1) and those with lower ratings in the other group (C2) (Figure 3).

The statistical power of these two combined analyses indicates that the PCA enables the videos to classify themselves in two groups (C1, high-quality videos, and C2, low-quality videos) without any information being lost on any of the variables considered in the entire study. Consequently, the quality of the videos can be analyzed across the two principal components, rather than having to do so across all the variables in the study because the PCAs have a high statistical relationship with the variables evaluated. This is a huge advantage when carrying out this kind of analysis, considering that no important information is lost through these two principal components because their level of relationship with the main variables that evaluate the quality of the videos is maximum, and *p*-values reveal a high, statistically significant relationship.

The statistical power of these combined analyses is such that it enables the videos’ classification, according to their quality, by considering two variables (principal components) that combine together the information from all the other variables analyzed in the study.

Health Institutions and Healthcare Professionals refer more rigorously to the correct terminology for these types of exercises than the rest of the sources, who refer to them as exercises (without specifying the term). Using appropriate terminology, so that patients know the conceptual distinctions, is necessary due to the increasing culturalization of society. People are willing to feel as if they are “decision-makers” and not only “patients”, so they demand a quota of self-resolution of their needs, with its advantages and disadvantages, such as self-taught training through the new online resources. To carry out this learning, it is necessary to use appropriate terminology for each type of situation [36] and thus participate in the development of the patient’s own decision-making capacities, with explanatory models that enable “experience” of the disease to be acquired. The way of narrating the disease is not only an academic finding, since it has practical significance in education, health promotion, and the development of institutional and professional devices [37].

Most of the videos did not indicate the need for extra material to carry out the exercises, which facilitates their implementation at home.

Regarding the indication of when to perform the exercises, most authors did not specify it, followed by a high percentage who recommended performing them post-surgery. The lowest percentage (3.8%) corresponds to those that indicated its pre-operative performance. The data obtained do not agree with studies such as that of Milios et al., who showed that a PFME program started prior to prostate surgery enhanced post-surgical measures of pelvic floor muscle function, reduced post-prostatectomy incontinence, and improved QoL outcomes related to incontinence [9].

Evidence shows that daily performance frequency is important, with two sets of PFM exercises per day as the most highly recommended, in accordance with the Glazener study, which recommends pelvic floor contractions twice per day [38], or the study of Kraemer and Ratamess, who reported greater improvements when participants exercised twice a day compared to only once per day [39]. After analyzing the videos, it was observed that 78.9% of the videos did not mention the frequency, and only 16.5% indicated that they were to be performed 3 times a day, as other authors also indicate [9].

The most appropriate exercises should be performed in three positions, sitting down, standing up, and supine positions, during all sessions [4,38]. However, most videos did not mention the position, while only 15% indicated the need to perform them in several positions.

Breathing during exercises is very important, and subjects should not hold their breath during pelvic floor activation, avoiding the Valsalva maneuver [4]. Despite this fact, in most of the videos, it was not mentioned (91.7%), or if it was, no indication was made of how to do it (5.3%). Only a minimum (3%) correctly explained how to do contractions during prolonged expiration [8].

Most authors indicate that activation of the superficial abdominal muscles should be reduced by means of visual and tactile pointers, and accessory muscles should not be used when performing exercises, as indicated by Gómez Lanza [40]. Despite this, 94% of videos did not include any information on this topic.

The indication on fast- and slow-twitch training was not represented in the videos either, since only 21.8% of videos recommended work on slow twitch and 9.77% did so on fast twitch, whereas it is necessary to work on each of these types [9].

### Limitations

Adhering to the principles of simulating what users see, no searches were conducted under incognito mode so as to avoid the influence of browsing history and geographical locations.

Although YouTube content changes over time, our analysis represents the state of the videos at one specific time [41].

Only videos directly accessed on YouTube upon searching for ‘Prostate cancer–exercises–pelvic floor’ were included in this study. External links from other medical-related websites were excluded in our analyses.

As a massive Internet-based platform, YouTube searches are continuously evolving as new videos are constantly being uploaded, viewed, and rated. Furthermore, our search was limited to the first 150 videos. As other studies have previously explained, our methodology was designed to replicate the average patient’s search attempt, since most Internet users do not look past the first 50 search results [35].

## 5. Conclusions

One of the main barriers to the success of any training program is adherence, and different methods are needed to ensure that users access the most appropriate ones. YouTube can be a strategy to achieve this. It is a solid and useful source of information, but a process of verification and validation of the information available on the web is necessary, as well as educational programs to enable society to access the most reliable information. YouTube is one of the most important development tools of the eHealth era because videos are a simple, attractive, and affordable source of information, with great dissemination capacity available to the population. The quality of the videos available on YouTube regarding the recommended exercises for pelvic floor in PC surgery, according to the scores obtained, is High.

Educational and Health Institutions, as well as Health Professionals, Government Health Authorities, and legislators, must be involved in the correct development of policies that improve the information available on the web in order to generate a positive impact on the healthy behavior of the population.

Future research should develop tools to make it easier for patients to identify the highest quality videos from a health perspective.

## Figures and Tables

**Table 1:** Descriptive statistics.

	Mean	±	SD	Median	Min	–	Max
Views	124,354	±	472,419	3678	14	–	3,484,686
Days Online	1777	±	1180	1597	220	–	4518
Views/day	138.30	±	788.31	2.67	0	–	8808
Likes	1082	±	4883	21.00	0	–	48,668
Dislikes	68.58	±	265.45	1.00	0	–	2195
Subscribers	95,039	±	343,699	3570	0	–	2,980,000
VPI	92.28	±	8.89	94.38	50.00	–	100.00
DISCERN 1	0.98	±	0.15	1.00	0	–	1
DISCERN 2	0.59	±	0.49	1.00	0	–	1
DISCERN 3	0.89	±	0.31	1.00	0	–	1
DISCERN 4	0.53	±	0.50	1.00	0	–	1
DISCERN 5	0.36	±	0.48	0.00	0	–	1
DISCERN Total	3.35	±	1.25	3.00	0	–	5
GQS	3.38	±	1.02	4.00	1	–	5

**Table 2:** DISCERN and GQS scores.

	DISCERN Score	GQS Score
Academic Institution	3.88	4.00
Media	2.79	2.96
NGO	4.33	4.00
Health Institutions	3.88	3.77
Non-Healthcare Professional	1.75	2.19
Healthcare Professional	3.45	3.45
Total	3.35	3.38
Africa	0.00	1.00
America	3.33	3.36
Asia	3.00	3.00
Australia	3.55	3.55
Europe	3.40	3.40
Total	3.35	3.38

**Table 3:** ICC intervals.

Interval	Reliability *
Less than 0.5	Poor
0.5–0.75	Moderate
0.75–0.90	Good
Greater than 0.90	Excellent

* Based on the 95% confidence interval.
